# Overexpression of spinach non-symbiotic hemoglobin in *Arabidopsis* resulted in decreased NO content and lowered nitrate and other abiotic stresses tolerance

**DOI:** 10.1038/srep26400

**Published:** 2016-05-23

**Authors:** Xuegui Bai, Juan Long, Xiaozhao He, Jinping Yan, Xuanqin Chen, Yong Tan, Kunzhi Li, Limei Chen, Huini Xu

**Affiliations:** 1 Faculty of Life Science and Technology, Kunming University of Science and Technology, Jingming South Street, Kunming, Yunnan 650224, P.R. China

## Abstract

A class 1 non-symbiotic hemoglobin family gene, *SoHb*, was isolated from spinach. qRT-PCR showed that *SoHb* was induced by excess nitrate, polyethylene glycol, NaCl, H_2_O_2_, and salicylic acid. Besides, *SoHb* was strongly induced by application of nitric oxide (NO) donor, while was suppressed by NO scavenger, nitrate reductase inhibitor, and nitric oxide synthase inhibitor. Overexpression of *SoHb* in *Arabidopsis* resulted in decreased NO level and sensitivity to nitrate stress, as shown by reduced root length, fresh weight, the maximum photosystem II quantum ratio of variable to maximum fluorescence (Fv/Fm), and higher malondialdehyde contents. The activities and gene transcription of superoxide dioxidase, and catalase decreased under nitrate stress. Expression levels of *RD22*, *RD29A*, *DREB2A*, and *P5CS1* decreased after nitrate treatment in *SoHb*-overexpressing plants, while increased in the WT plants. Moreover, *SoHb*-overexpressing plants showed decreased tolerance to NaCl and osmotic stress. In addition, the *SoHb*-overexpression lines showed earlier flower by regulating the expression of *SOC*, *GI* and *FLC* genes. Our results indicated that the decreasing NO content in *Arabidopsis* by overexpressing *SoHb* might be responsible for lowered tolerance to nitrate and other abiotic stresses.

Nitrogen (N) is one of the essential mineral nutrients for plants and a large amount of N was added into the soil. However, only 30–50% of the applied fertilizer N is actually captured by crops, and the left part can subsequently leach and contaminate ground water^1^. Over-utilization of N fertilizer has resulted in secondary salinization in Chinese greenhouse. The excessively accumulated anion ion in soil of greenhouse is nitrate (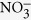)^2^,^3^. Excess nitrate inhibited the growth of vegetables^4^. Secondary salinization is thought to be one of the most important factors that seriously limit the sustainable development of protected agricultural production in China.

Hemoglobins (Hbs) are ubiquitous proteins found in all kingdoms of life^5^. In plants, three types of hemoglobins exist: symbiotic, non-symbiotic, and truncated hemoglobins. Non-symbiotic hemoglobins (nsHbs) can be further divided into class 1 (nsHb1, GLB1) and class 2 (nsHb2, GLB2) proteins that exhibit different oxygen-binding properties and gene expression patterns^6^,^7^. *NsHb* genes have been isolated from a number of both dicot and monocot species, such as, *Arabidopsis*, barley, casuarina, rice, trema, soybean, tomato^8^, oak^9^, and maize^10^. *NsHb1* is involved in various biotic and abiotic stress responses. For example, *nsHb1* genes have been reported to be up-regulated by hypoxic, osmotic, and high salt^6^,^10^,^11^, short-term waterlogging^12^, treatments with salicylic acid, methyl jasmonic acid, ethylene, and H_2_O_2_^13^, deficiency of phosphorus, potassium, and iron^8^, as well as dual culture with specific microsymbionts^14^. *NsHb1* genes were also strong induced by nitrate, nitrite, or nitric oxide (NO)^15^,^16^.

NO is an important signaling molecule involved in the physiological processes of plants^17^. Multiple physiological functions of NO in plants have been reported, including the stimulation of seed germination, suppression of floral transition, mediation of stomatal movement and modulation of plant growth, development, plant maturation and senescence, as well as abiotic and biotic stresses response^18^,^19^,^20^,^21^,^22^. There are several potential pathways for generating endogenous NO in plants. Among them, nitrate reductase (NR) and nitric oxide synthase (NOS) are two key enzymes for NO synthesis in plants^23^. NsHb have been shown to be involved in scavenging of NO molecules^24^. NsHb1 exhibit an extremely high affinity for NO^25^,^26^. Alfalfa (*Medicago sativa*) *Mhb1* has a potential link to the NO signaling pathway^27^. Studies of transgenic *Arabidopsis* and *alfalfa* root cultures overexpressing *nsHb1* genes suggest that *nsHb1* may be involved in NO-mediated pathways of hypoxic or early-growth states^28^,^29^. NsHb was also shown to be involved in NO metabolism in barley, as ectopic expression reduced the amount of NO released during hypoxia^24^.

Spinach (*Spinacia oleracea* L.) is one of the most popular leafy vegetables grown in Chinese greenhouses, particularly in the Dianchi River division of Yunnan province. It is also an excellent source of dietary vitamins and minerals such as calcium and magnesium. In our previous work, a subtractive suppression hybridization (SSH) library of spinach roots under nitrate stress was constructed and several nsHb ESTs were found^4^. The function of *SoHb* in response to excess nitrate stress has not been investigated yet. Here, we report the molecular cloning and functional characterization of *SoHb* from spinach. Expression of the *SoHb* gene was analyzed by real-time quantitative RT-PCR (qRT-PCR) in spinach grown under nitrate or other abiotic stresses. Ectopic expression in *Arabidopsis* conferred decreased tolerance to nitrate and other abiotic stresses.

## Results

### Cloning and characterization of a spinach hemoglobin gene

Based on the suppression subtractive hybridization (SSH) assay in our previous study^4^, a 409 bp EST representing a putative hemoglobin gene was strongly induced under nitrate stress. By 5′RACE and 3′RACE cDNA cloning, a 124 and 265 bp cDNA was generated, respectively. By comparing and aligning the EST sequence of hemoglobin, the 5′RACE and 3′RACE products, the full-length cDNA of the *SoHb* gene was obtained, and it was subsequently confirmed by sequencing. The full-length cDNA of *SoHb* (GenBank Accession No. KC142173) was 667 bp and contained a single ORF of 504 bp, flanked by stretches of 51 and 112 bp at the 5′ and 3′ untranslated regions, respectively (Figure S1). The ORF encoded a predicted polypeptide of 167 amino acid residues, with a predicted molecular mass of 18.71 kDa and pI of 8.97.

**Figure 1:**
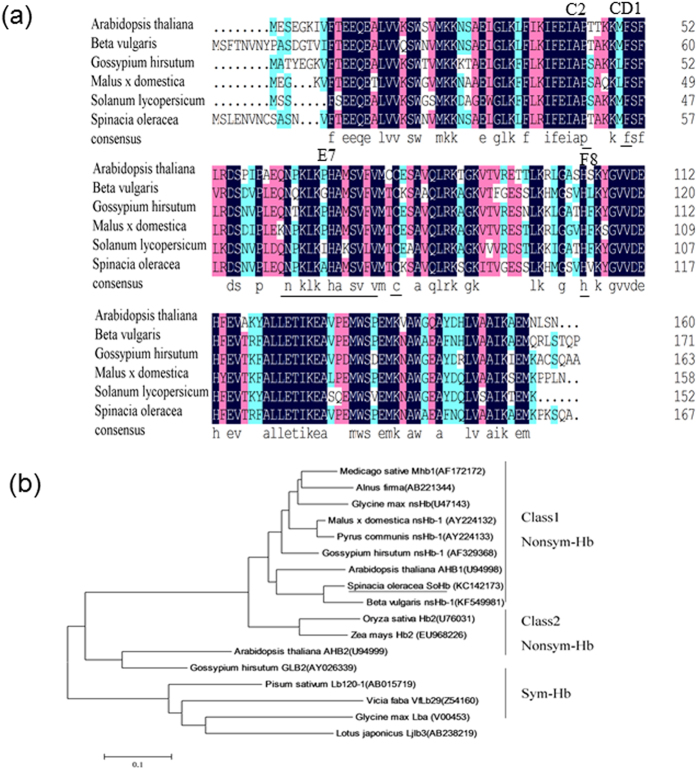
Analysis of the deduced amino acid sequence of SoHb. (**a**) Multiple sequence alignment of SoHb. Alignment of the predict SoHb protein with other Hb proteins from different plants. Conserved residues of heme- and ligand-binding (distal (E7) and proximal (F8) His residues, Phe (CD1), Pro (C2)) are underlined. Cys residues are also underlined. The protein sequences shown in the diagrams were listed in the GenBank database under the following accession numbers: *Spinacia oleracea* (Acc. No. KC142174); *Beta vulgaris* (Acc. No. KF54998); *Arabidopsis thaliana* (Acc. No. AK227823); *Malus*×*domestica* (Acc. No. AY224132); *Gossypium hirsutum* (Acc. No. AF329368); *Solanum lycopersicum* (Acc. No. NM001247569). (**b**) Phylogenetic analysis of SoHb together with the other plant nsHbs. The phylogenetic tree for Hb from different plant species were constructed on the basis of the multiple alignments of deduced amino acid sequences performed using ClustalW with default parameter settings and computed using the NJ method in the MEGA 6. The database accession numbers were indicated in parentheses after plant names.

Sequence alignment, using DNAman, of the predicted amino acid residues of SoHb with different members of the Hb family clearly established that SoHb had similarity with other Hb proteins. SoHb showed the highest identity with *Beta vulgaris* of 84.21%. SoHb showed identity with *Gossypium hirsutum*, *Malus x domestica*, *Arabidopsis thaliana*, *Solanum lycopersicum* of 75.00%, 73.05%, 71.86%, 66.47%, respectively (Fig. 1a). The SoHb deduced protein contained all conserved amino acid of plant Hbs. These include the CD1 phenylalanine, C2 proline and F8 proximal histidine residues needed for heme binding, and the E7 distal histidine which is involved in ligand binding^30^. A cysteine residue, found in most known plant nonsymbiotic Hbs, was also present in SoHb.

To clarify the relationships between SoHb with the other Hb proteins, the phylogenetic tree was generated. The result showed that SoHb fell into the class 1 family of non-symbiotic hemoglobin (Fig. 1b).

### Expression of *SoHb* in spinach under nitrate and other abiotic stresses

**Figure 2:**
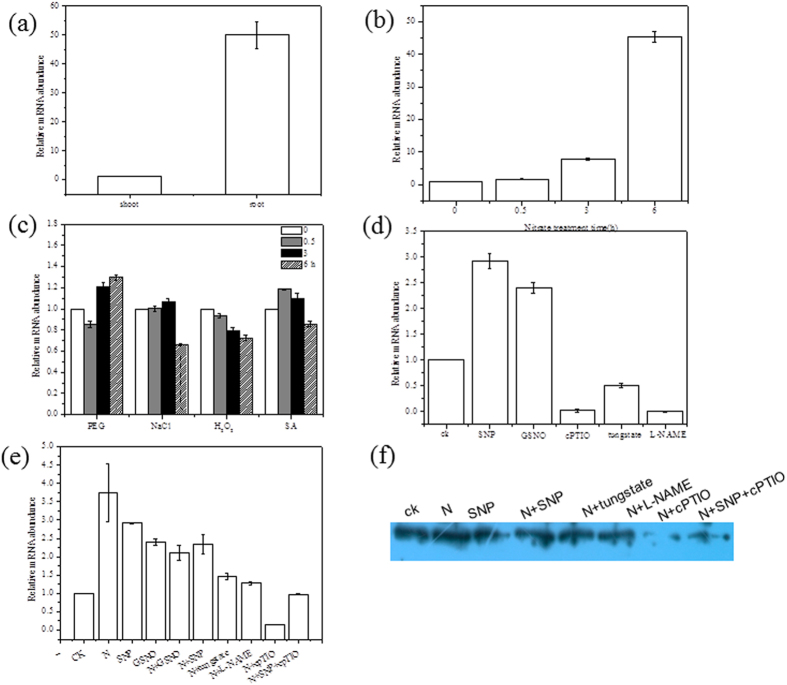
Expression characteristics analysis of *SoHb*. **(a)** Organ-specific expression of *SoHb* by qRT-PCR. **(b)** Expression of *SoHb* under 100 mM nitrate treatment for 0, 0.5, 3 and 6 h. **(c)** Expression of *SoHb* under PEG, NaCl, SA and H_2_O_2_ stresses. **(d)** Expression of *SoHb* under NO donor, No scavenger and inhibitor treatment. **(e)** Expression of *SoHb* under nitrate stress supplemented with NO donor, No scavenger and inhibitor. **(f)** Western blot analysis of SoHb under nitrate stress supplemented with NO donor, No scavenger and inhibitor. Values are means ± S.E. based on three replicates.

Expression profiling of the *SoHb* was carried out by qRT-PCR in spinach grown in hydroponic cultures. Organ specific study showed that *SoHb* expressed in both root and shoot, and the expression in root was higher than in shoot (Fig. 2a). qRT-PCR was also conducted to examine the expression pattern of *SoHb* in spinach under 100 mM nitrate treatment for 0, 0.5, 3, and 6 h. The result showed that the *SoHb* expression level increased gradually from 0.5 to 6 h-treatment, and reached highest expression in 6 h. The expression increased 45.27-fold after 6 h-treatment (Fig. 2b).

To examine whether *SoHb* was induced by other abiotic stresses, spinach root was treated with various inducers including PEG, NaCl, and global signaling molecules SA, and H_2_O_2_. As shown in Fig. 2c, when treated with PEG, the transcripts of *SoHb* decreased after 0.5-h treatment and then increased after 3, and 6-h treatment. When treated with NaCl, the expression levels first increased gradually and then decreased after 6-h treatment. The transcripts level of *SoHb* decreased gradually with the increasing H_2_O_2_ treatment time. After SA treatment, the *SoHb* expression reached highest expression in 0.5 h, then the expression decreased gradually.

Since Hb is known to be involved in NO detoxification, SNP (an NO donor), GSNO (an NO donor), cPTIO (an NO scavenger), tungstate (NR inhibitor), and L-NAME (NOS inhibitor) were applied to the solution. The transcript level of *SoHb* was strongly induced by 100 μM SNP and 100 μM GSNO. When plants were treated with 100 μM cPTIO, the transcript level of *SoHb* was repressed strongly. When treated with tungstate and L-NAME, the *SoHb* expression was decreased (Fig. 2d). The expression levels of *SoHb* gene and protein were analyzed by qRT-PCR and western blot after SNP, cPTIO, tungstate, and L-NAME were added into the nitrate solution (Fig. 2e,f). The result showed that after adding SNP and GSNO to the nitrate solution for 24 h, compared with the nitrate or SNP and GSNO treatment alone, the *SoHb* gene expression level were decreased. When tungstate, L-NAME, or cPTIO were added into the nitrate solution, the *SoHb* gene and protein level were also inhibited.

To understand the relationship between the existence of NO and the expression of *SoHb*, the accumulation of NO in root tissues were analysed by confocal laser microscope using NO sensitive dye, 3-amino, 4-aminomethyl-2′,7′-difluorescein, diacetate (DAF-FM DA) as a NO detector. As shown in Figure S2, the intensity of fluorescence was dramatically increased by the addition of SNP or nitrate. When SNP was added into the nitrate solution, the NO fluorescence was lower than the SNP and nitrate treatment alone, but was higher than the control. When the root were treated with tungstate, L-NAME, or cPTIO, the intensity of the fluorescence was decreased compared with the control, but was still detectable.

### Overexpression of *SoHb* decreased the NO content in *Arabidopsis*

To investigate the biological function of *SoHb*, transgenic *Arabidopsis* plants were created under the control of the CaMV 35S promoter. The transgenic plants carrying *SoHb* were detected by PCR after the first screening with 30 mg/mL kanamycin. 20 kanamycin resistant putative transformants were obtained. Using specific primers, the *SoHb* gene was detected in the transgenic plants, while this gene was not detected in the WT plant (Figure S3a). The RT-PCR results showed that these kanamycin-resistant plants had significantly higher mRNA expression than the WT plants (Figure S3b). Western blot analysis revealed the presence of strong positive protein signals corresponding to SoHb in transgenic plants, while no signal was detected in WT plant (Figure S3c). Two lines (Hb-1, Hb-3) were selected for further analysis.

**Figure 3:**
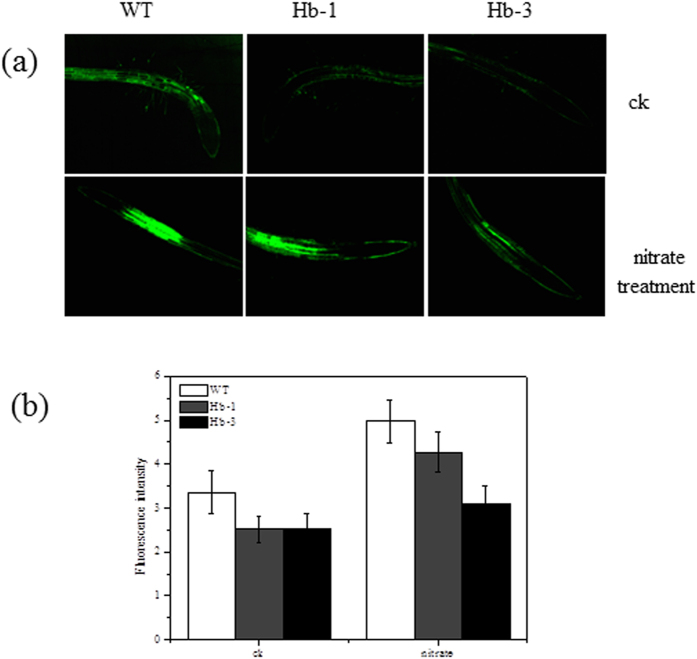
The NO fluorescence in *SoHb* transgenic *Arabidopsis* grown in MS medium with or without nitrate stress treatment. (**a**) Endogenous NO in *Arabidopsis* root was visualised using the DAF-FM DA with a confocal lasers scanning microscope system. (**b**)Average fluorescence intensity levels of root tips of WT and transgenic lines. The experiment was replicated three times with similar results. Bars = 100 μM.

The NO contents of the two transgenic lines were assayed by the DAF-FM DA staining. The results indicated that two transgenic lines showed lower NO contents in root compared with the WT. When the *Arabidopsis* plants were treated with100 mM nitrate stress, the NO accumulation in all the tested lines were found, but the increase in transgenic lines was dramatically less than that of the WT, especially in the Hb-3 line (Fig. 3).

**Figure 4:**
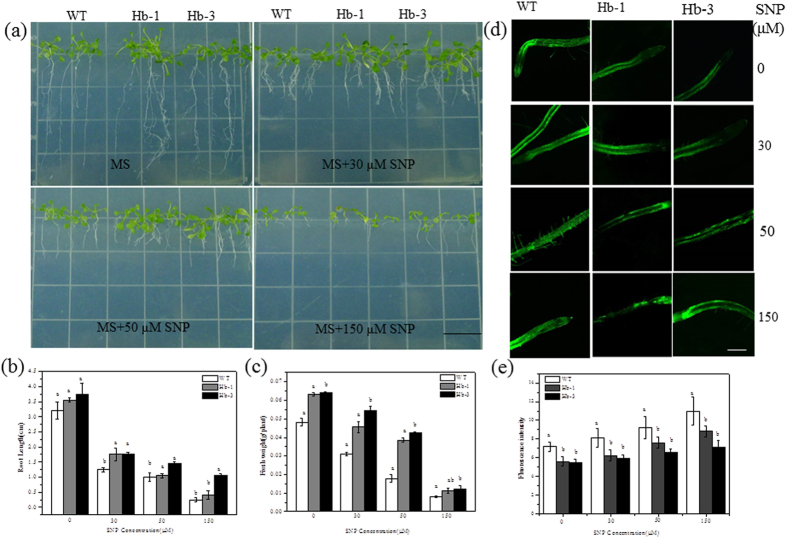
Effect of excess SNP on the growth of *SoHb* transgenic *Arabidopsis* for 7 days. Seedling morphology (**a**), root length (**b**), fresh weight (**c**) of the WT and transgenic lines grown on MS supplemented with 0, 30, 50, and 150 μM SNP. Bars = 1 cm. **(d)** The NO content of WT and transgenic plants under excess NO stress treatment. WT was wild type Columbia *Arabidopsis*; Hb-1 and Hb-3 were two homozygous transgenic *Arabidopsis* lines. (**e**) Average fluorescence intensity levels of root tips of WT and transgenic lines grown under different concentration of SNP. The experiment was replicated three times with similar results. Bars = 100 μM.

Previous work provided compelling evidence that GLB1 proteins were primarily involved in modulating NO concentrations^31^. We hypothesized that endogenous NO in transgenic plants under nitrate stress might be reduced by constant *SoHb* expression, which can act as an NO scavenger. To test the hypothesis, seedling growth assay was performed on MS medium supplemented with 0, 30, 50, and 150 μM SNP. The root growth of both WT and transgenic plants was inhibited in the medium supplemented with 30 to 150 μM SNP (Fig. 4). However, the inhibition in WT plants was more than that in the transgenic plants. The fresh weight and root length decreased less in transgenic plants, especially in the Hb-3 lines. The seedling growth assay was also performed on MS medium supplemented with 0, 30, 50, and 150 μM GSNO. The root length and fresh weight were also higher in *SoHb* transgenic plants than the WT plants (Figure S4).

The NO contents were also analysed by confocal laser microscope. Under normal conditions, the NO florescence signals were weaker in transgenic root than the WT. The NO contents were increased in all the plants root with the increasing of SNP concentration. However, NO florescence signals of the transgenic plants were weaker than the WT plants, especially in the Hb-3 line root (Fig. 4d).

### Overexpression of *SoHb* decreased the nitrate stress tolerance of *Arabidopsis*

**Figure 5:**
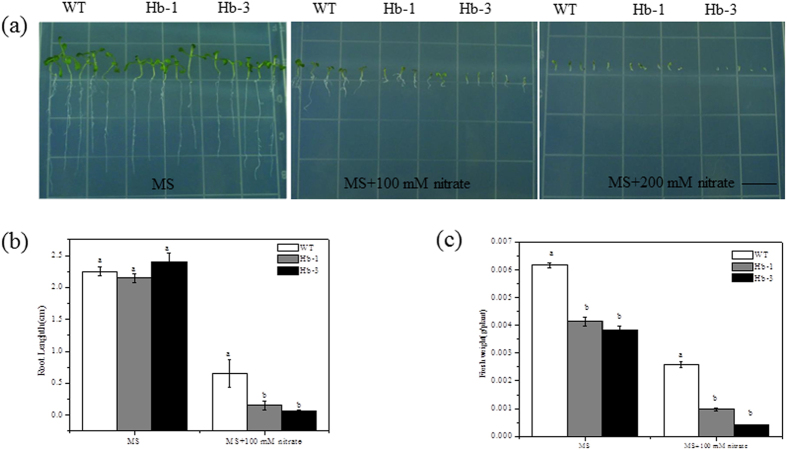
Effect of excess nitrate stress on the growth of *SoHb* transgenic *Arabidopsis*. Seedling morphology (**a**), root length (**b**), fresh weight (**c**) of the WT and transgenic lines grown on MS medium supplemented with 100 and 200 mM nitrate for 7 days.

To analyze the nitrate stress tolerance, the germinated transgenic plants were planted in the MS medium with 100 and 200 mM nitrate. As shown in Fig. 5, overexpression of *SoHb* resulted in reduction of nitrate stress tolerance. The transgenic plants have significantly shorter root and lower fresh weight than the WT plant under 100 mM nitrate treatment (*P* < 0.05).

To investigate if exogenous SNP can alleviate the inhibition of root growth of the nitrate-stressed *Arabidopsis*, 5 μM SNP was added into the MS medium supplemented with 100 mM nitrate. The result showed that the root length of transgenic plants were longer than the nitrate-stressed plants after adding SNP (Figure S5).

**Figure 6:**
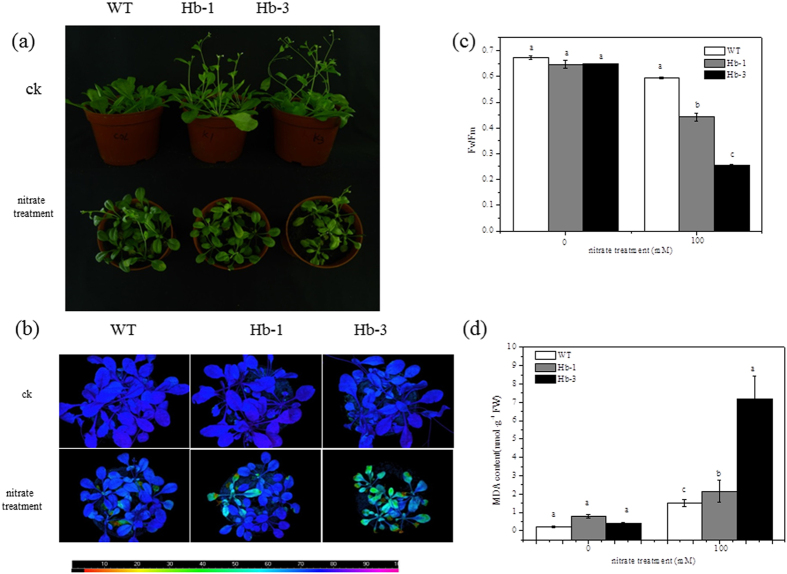
Effects of nitrate stress on the tolerance of adult transgenic seedlings. **(a)** Morphology of adult plants of transgenic *Arabidopsis* after nitrate treatment. **(b)** Images of Fv/Fm (bottom); F0 (top) was used as the control. The pseudocolor code depicted at the bottom of the image ranges from 0 (black) to 1.0 (purple). **(c)** Average Fv/Fm values. **(d)** The lipid peroxidation analysis of WT and transgenic plants after nitrate stress treatment. Plants were treated with 100 mM nitrate solution for 15 days. The experiment was replicated three times with similar results. Means denoted by different letters show significant differences at *P* < 0.05 according to Duncan test.

To further demonstrate the tolerance of nitrate stress, 2-week-old T3 seedlings were irrigated every day with nitrate solution for 15 d. The phenotypic effects of the salt treatment were shown in Fig. 6a, the transgenic plants showed more seriously chlorotic symptoms than the WT plants. To determine the effects of nitrate on photosynthetic efficiency, we compared the maximum photochemical efficiency of PSII in the dark-adapted state (Fv/Fm). There was no difference of Fv/Fm between WT and the transgenic lines under normal conditions. Excess nitrate resulted in significant decreases in Fv/Fm of the transgenic plants compared with the WT plants (Fig. 6b,c). The low value of Fv/Fm (0.44, 0.25) in Hb-1 and Hb-3 lines after treatment indicated that the transgenic plants suffered more severe damage than the WT plants (0.59).

The MDA contents, as an indicator for the degree of oxidative stress, were analysed. The MDA contents after nitrate stress treatment were elevated remarkably in all tested lines. However, it was clear that MDA content of WT (1.54 nmol g^−1^ FW) was significantly lower than those of the Hb-1 and Hb-3 transgenic lines (2.15, 7.20 nmol g^−1^ FW) (Fig. 6d), indicating that overexpression of *SoHb* in the *Arabidopsis* plants confers decreased detoxification ability.

### The physiological and molecular response of *SoHb* transgenic plants to nitrate stress

**Figure 7:**
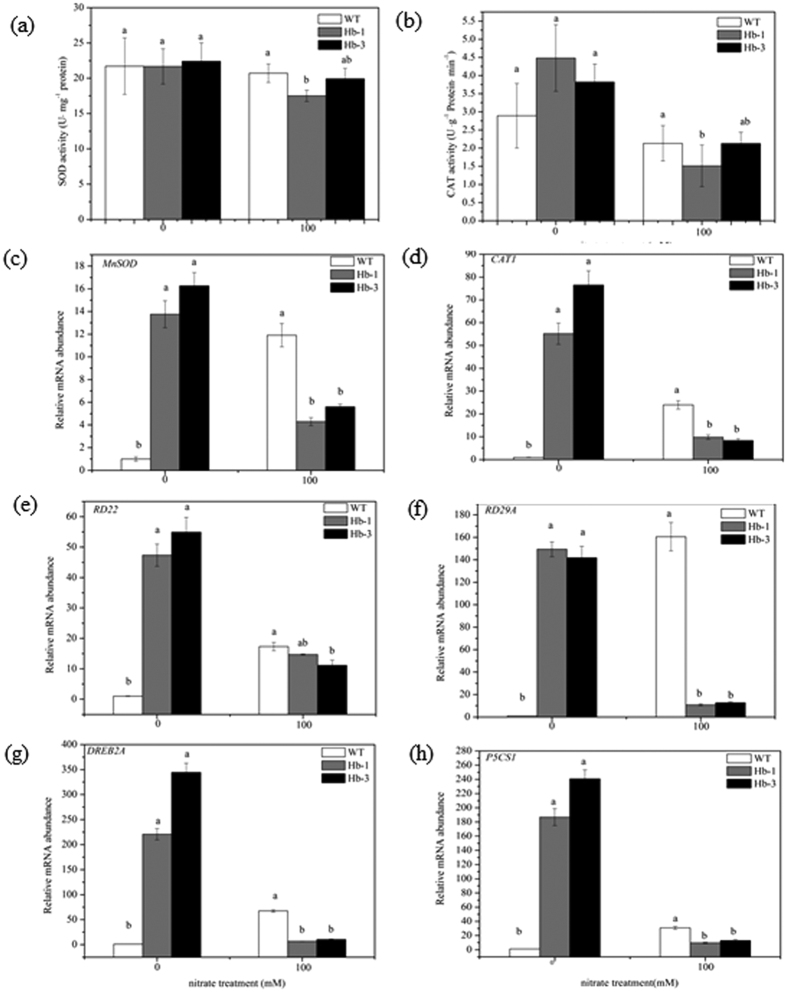
The physiological and molecular response of *SoHb* transgenic plants to nitrate stress. (**a–d**) The antioxidant enzyme activities and relative transcript levels of SOD and CAT of WT and transgenic plants. (**e–h**) The expression of stress-induced genes of *RD22*, *RD29A*, *DREB2A*, and *P5CS1* in WT and transgenic plants assayed by qRT-PCR. Plants were treated with 100 mM nitrate solution for 15 days. The experiment was replicated three times with similar results. Means denoted by different letters show significant differences at *P* < 0.05 according to Duncan test.

To elucidate the physiological mechanisms underlying the decreased stress tolerance, activities of SOD and CAT in the transgenic lines and WT were analyzed after the nitrate treatment. As shown in Fig. 7, before nitrate treatment, there was no significant difference of SOD and CAT activities between the transgenic and WT plants. After nitrate treatment, the activities of SOD, and CAT decreased in the transgenic and WT plants, especially in the transgenic lines. After nitrate treatment, the SOD activities of Hb-1, Hb-3 and WT decreased by 21.72%, 11.11%, 4.61%, respectively; and the CAT activities of Hb-1, Hb-3 and WT decreased by 66.07%, 44.50%, 26.30%, respectively.

To partly confirm the results of gene expression at transcriptional levels, the expression of *MnSOD* and *CAT1* were analyzed by qRT-PCR assay. Before the nitrate stress, mRNA levels of *MnSOD* and *CAT1* of the transgenic lines were significantly higher than that of the WT. After the nitrate treatment, the mRNA abundance of the WT plants displayed the higher transcription level of *MnSOD*, and *CAT1*, whereas their transcriptions were decreased in the two transgenic lines.

To elucidate the further role of *SoHb* in stress tolerance, we examined the effects of *SoHb* on the transcript levels of several stress inducible genes including *RD22* (*Responsive to Dehydration 22*), *RD29A*, *DREB2A* (*Dehydration-Responsive Element-Binding Factor 2A*), *P5CS1* (*delta1-Pyrroline-5-Carboxylate Synthetase 1*). Under normal conditions, the expression levels of *RD22*, *RD29A*, *DREB2A*, *P5CS1* were strongly higher than that of the WT (*P* < 0.05). In the presence of nitrate stress treatments, the expression of all of these stress-responsive genes increased in the WT plants, but decreased in the two transgenic lines (Fig. 7).

### Overexpression of *SoHb* decreased the NaCl and osmotic stress tolerance of *Arabidopsis*

**Figure 8:**
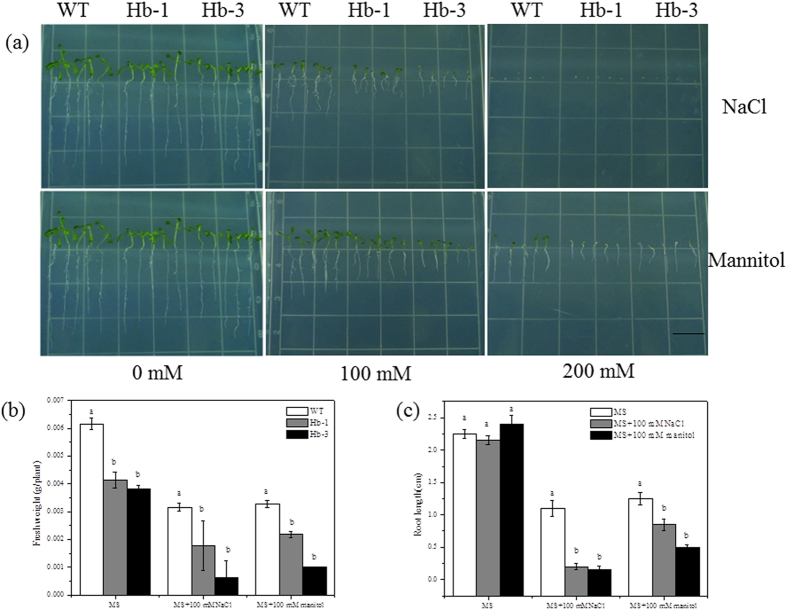
Effect of NaCl and osmotic stress on the growth of *SoHb* transgenic *Arabidopsis* for 7 days. Seedling morphology (**a**), root length (**b**), fresh weight (**c**) of the WT and transgenic lines grown on MS supplemented with 0, 100, and 200 mM NaCl and mannitol. WT was wild type Columbia *Arabidopsis*; Hb-1 and Hb-3 were two homozygous transgenic *Arabidopsis* lines. The experiment was replicated three times with similar results. Bars = 1 cm.

To further analyze the other abiotic stresses tolerance of the transgenic plants, the germinated transgenic plants seeds were grown in the MS medium with 100 and 200 mM NaCl and mannitol, mimics the salt and osmotic stress. As shown in Fig. 8, overexpression of *SoHb* in transgenic plants resulted in reduction of the stress tolerance of NaCl and mannitol. After NaCl treatment, the fresh weight of Hb-1, Hb-3, and WT plants decreased by 57.01%, 69.10% and 48.73%, respectively; and the root length of Hb-1, Hb-3, and WT plants decreased by 86.72%, 93.81%, and 51.50%. After the osmotic stress, the fresh weight and root length of Hb-1, Hb-3, and WT plants decreased by 47.31%, 83.23%, 46.84%, and 62.22%, 79.21%, 44.45%, respectively, indicating that overexpression of *SoHb* decreased the salt and osmotic stress tolerance.

### Influence of *SoHb* overexpression on plant development

**Figure 9:**
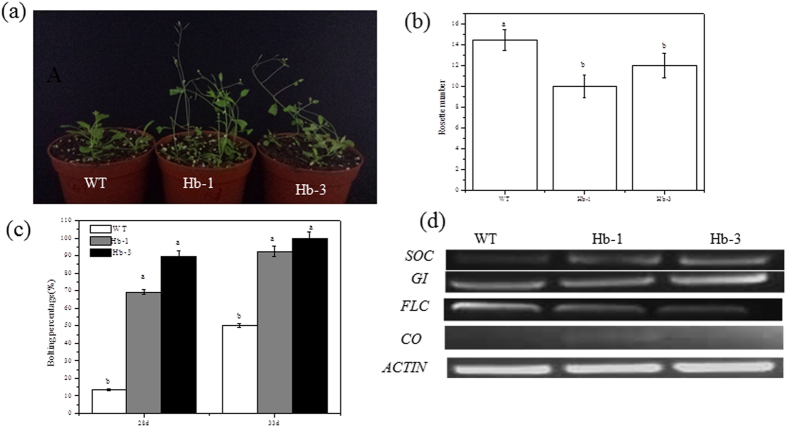
Overexpression *SoHb* transgenic plants showed earlier flower time phenotype. (**a**) *SoHb* transgenic lines flowered earlier. WT and *SoHb* transgenic lines were grown in soil under 16 h light/8 h dark cycles and were photographed after 28 d of growth. (**b**) Rosette leaf number of WT and transgenic *SoHb* lines during flowering (n > 30 plants). (**c**) The percentage of flower plants of WT and transgenic *SoHb* lines (n > 30 plants). (**d**) *SOC*, *FLC*, *CO* and *GI* expression in WT and transgenic *SoHb* lines assayed by semi-quantitative RT-PCR. All experiments were repeated at least three times. Means denoted by different letters show significant differences at *P* < 0.05 according to Duncan test.

It has been documented that NO can repress floral transition in *Arabidopsis*^32^. Thus, the flowering time of WT and *SoHb* overexpression transgenic lines was investigated. Our results indicated a decrease in the rosette leaf number of *SoHb* transgenic plants, while the days to flowering were earlier than the WT plants (Fig. 9a). In 28 d, the bolting percentage of Hb-1, Hb-3, and WT plants were 13.52%, 69.21%, and 89.70%, respectively (*P* < 0.05). Four key flowering genes *CO* (*CONSTANTS*), *SOC* (*SUPPRESSOR OF OVEREXPRESSION OF CONSTANS*), *FLC* (*FLOWEREING LOCUS C*), and *GI* (*GIGANTEA*) were assayed for their expression by semi-quantitative RT-PCR (Fig. 9d). The result showed that the *SoHb* transgenic plants up-regulated *SOC* and *GI* expression and down-regulated *FLC* expression, while the expression of *CO* was not significant affected, in accordance with their earlier flowering phenotype.

Besides, NO also affect the root growth and later root development. *SoHb* overexpressing transgenic plants have less lateral root than the WT plants. The average number of lateral root of the WT plants was 7.31, while the average lateral root number of the transgenic plants Hb-1 and Hb-3 were reduced to 5.02 and 4.05, respectively (Figure S6).

## Discussion

Plant Hbs exist ubiquitously in the plant kingdom. In this study, we identified a gene encoding Hb in spinach root, which was designated as *SoHb*. The analysis of the *SoHb* expression pattern in various plant organs showed that the gene was strongly expressed in root as compared with the shoot, suggesting a more prominent role for *SoHb* in root. Hbs play important roles in various physiological processes in plants. A class 1 hemoglobin gene of *Arabidopsis thaliana*, *AtHB1*, was highly induced under hypoxia, by sucrose addition^6^ and nitrate addition^33^. *GhHb1* expression was induced by SA, H_2_O_2_ and NO^13^. In this study, *SoHb* was induced by excess nitrate. PEG and NaCl also induced the expression of *SoHb*, suggesting that it may be involved in various defense pathways. Besides, the *SoHb* expression was induced in NO-promoting conditions, such as nitrate stress, by a NO donor (SNP), and the expression was repressed when the seedlings were treated with a NO scavenger, NR and NOS inhibitor. The transcripts of *OsnsHB1* gene were also induced by NO in cultured rice cells^16^. These results suggested that *SoHb* expression corresponds to the cellular concentration of NO and might be involved in the modulation of NO levels in spinach.

NO is an important signaling molecule with diverse physiological functions in plants^34^,^35^,^36^, but its exact role in the response of plants to nutritional stress is still under evaluation. NO signaling is a key component of the root growth response to nitrate in *Zea mays* L^37^. Inhibition of root elongation in maize by high external nitrate is likely to result from a reduction of nitric oxide synthase-dependent endogenous NO levels in maize root apical cells^38^. NO is produced by nitrate reductase (NR) as an early response to nitrate supply and that the coordinated induction of nsHbs could finely regulate the NO steady-state^39^,^40^. SNP markedly enhanced endogenous NO levels in root apices grown in high nitrate, but they had no effect on endogenous NO levels in maize root apical cells grown in low-nitrate solution^38^. In our study, after 24-h nitrate stress or SNP treatment, the NO content increased in the spinach and the *Arabidopsis* roots, indicating that NO was involved in the excess nitrate stress response in spinach or *Arabidopsis*.

Transgenic maize cells with reduced levels of nsHb1 proteins produce more NO than WT cells^25^, whereas cultured alfalfa root cells with an increased level of barley nsHb1 accumulate less NO than the control cells^29^. Endogenous NO increased dramatically in the salt-treated WT plants but not in the *TrHb* transgenic plants^41^. GhHb1-transgenic *Arabidopsis* seedlings were more tolerant to exogenous NO and contained lower levels of cellular NO than the WT control^13^. In our experiment, the endogenous NO content in *SoHb*-overexpression plants was lower compared with the WT plants. Upon treatment with SNP, the seedling growth was less retarded in overexpressing *SoHb* transgenic lines compared with the WT seedlings.

Recent studies have reported the functional analysis of class 1 *nsHb* in transgenic plants. A transgenic *Arabidopsis* culture overexpressing *Arabidopsis* Hb showed resistance to hypoxia^28^. Ectopic expression of *ZmHb* in transgenic tobacco has enhanced tolerance to submergence, salinity and osmotic stresses^10^. Ectopic overproduction of *GhHb1* in *Arabidopsis* led to constitutive expression of the defense genes PR-1 and PDF1.2, and conferred enhanced disease resistance to *Pseudomonas syringae* and tolerance to *V. dahliae*^13^. The *Arabidopsis glb1* mutant line has increased resistance to pathogens, which was reduced in 35S-GLB1 compared to Col-0^42^. In our study, under excess nitrate, NaCl, and osmotic stress, *SoHb*-overexpression plants grew worse than WT plants, suggesting that *Arabidopsis* plants with increased levels of *SoHb* were more sensitive to these abiotic stresses. This might because NO was not available in sufficient amounts to stimulate the salt tolerance signal transduction pathway. Removing NO produced an abnormal defense mechanism in the transgenic plants^41^.

Increased salt stress results in enhanced accumulation of reactive oxygen species (ROS) that are harmful for plant cells in high concentrations and subsequently led to lipid peroxidation. To avoid oxidative damage, plants are equipped with efficient antioxidant systems that can protect them from deleterious effects of ROS. Previous studies have shown that the activities of antioxidant enzymes, such as superoxide dismutase (SOD), and catalase (CAT) are directly correlated with stress tolerance in plants, and an augmented ability to scavenge ROS has been observed in plants that grow in sublethal levels of stress^43^. It has been reported that NO protects plant cells against oxidative stress by reducing ROS accumulation under salt stress^44^,^45^,^46^. Under stress conditions, NO regulates antioxidant enzymes at the level of activity and gene expression, which can cause either enhancement or reduction of the cellular redox status^47^. Rapid NO accumulation in response to strong stress stimuli was occasionally linked to inhibition of antioxidant enzymes and a subsequent rise in hydrogen peroxide levels^47^. In our study, the lipid peroxidation levels of the overexpression *SoHb* transgenic plants were higher than that of the WT plants, suggesting that the transgenic plants were damaged seriously by nitrate stress treatment. The activities and the transcription of *MnSOD* and *CAT1* in S*oHb*-overexpression plants decreased more than that of WT plants after nitrate treatment, indicating that S*oHb*-overexpression plants decreased the ROS scavenging ability, which might be responsible for more serious oxidative stress damage.

High salinity usually induces the expression of stress-responsive genes. *RD22*, *RD29A*, and *DREB2A* are known to be involved in responses to drought and salt stress^48^,^49^,^50^. Proline accumulation is benifical for stress tolerance^51^, and *P5CS1* encodes a key enzyme in the biosynthesis of proline^52^,^53^. In our study, overexpression of *SoHb* plants increased the expression of *RD22, RD29A*, *DREB2A*, *P5CS1* under normal growth conditions. However, the expression of these genes decreased under nitrate stress, indicating that the differences in salt tolerance between the WT and transgenic plants might be partly due to the decreased expression of *RD22, RD29A*, *DREB2A*, *P5CS1*.

NO suppressed *CO* and *GI* gene expression and enhanced *FLC* expression, which indicated that NO regulates the photoperiod and autonomous pathways^32^. *GI* integrates cellular signals from light sensory transduction and the circadian clock and activates *CO*^54^. *CO* acts as a mediator between the circadian clock and the control of flowering^55^. *SOC* promotes flowering of *Arabidopsis*, and *soc1* mutant is a suppressor of flowering^56^. *FLC* is a central floral repressor gene acting in the control of vernalization or autonomously induced flowering^57^. Lines with *GLB1* silencing had a significant delay of bolting and after bolting, shoots reverted to the rosette vegetative phase by formation of aerial rosettes at lateral meristems. Lines with overexpression of *GLB1* or *GLB2* bolted earlier than wild type plants^58^. Here, the expression of these genes was also regulated in transgenic *SoHb* plants. 28-day-old *SoHb* overexpression plants had a higher expression of *SOC* and *GI*, while lower *FLC* expression level than WT plants, which flowered earlier, consistent with previous results showing that NO represses the photoperiod and autonomous floral pathways through *FLC*^32^, suggesting that non-symbiotic plant hemoglobin controls bolting by scavenging the floral transition signal molecule, NO.

## Methods

### Plant growth and stress treatments

Spinach seeds (*Spinacia oleracea* L. cv. Chaoji) were germinated on moisture filter paper in an incubator at 28 °C. After 15 days, batches of 20 seedlings were transferred to a plastic tank (40 × 30 × 12 cm) with 10 L of nutrient solution containing Ca (NO_3_)_2_ 2.5 mM, KNO_3_ 5 mM, KH_2_PO_4_ 0.78 mM, MgSO_4_ 2 mM, H_3_BO_3_ 29.6 μM, MnSO_4_ 10 μM, Fe-EDTA 50 μM, ZnSO_4_ 1.0 μM, H_2_MoO_4_ 0.05 μM, CuSO_4_ 0.95 μM. pH of the nutrient solution was adjusted to 6.0–6.5 by addition of 98% (w/w) H_2_SO_4_. The experiment was carried out in a greenhouse of Kunming University of Science and Technology. The light period was about 12 h, and the air temperature was 20–28 °C during the day and 13–18 °C during the night.

For excess nitrate, NaCl, dehydration, hydrogen peroxide (H_2_O_2_) and salicylic acid (SA) treatments, 4-week-old seedlings were transferred into solutions containing either 100 mM nitrate (KNO_3_ and Ca(NO_3_)_2_ provide the same mol of 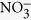), 10% polyethylene glycol (PEG) 6000 (w/v), 150 mM NaCl, 1 mM H_2_O_2_, 1 mM SA for 0, 0.5, 3, and 6 h. Shoot and root of spinach seedlings were taken after different treatments, immediately frozen in liquid nitrogen and stored at −80 °C until use.

For NO treatment, 4-week-old spinach seedlings were treated with the NO donor, sodium nitroprusside (SNP, 100 μM) and *S*-nitrosoglutathione (GSNO, 100 μM), the NO scavenger 2-(4-carboxyphenyl)-4,4,5,5- tetramethylimidazoline-1-oxyl-3-oxide (cPTIO, 100 μΜ), the NR inhibitor tungstate (100 μΜ), or the NOS inhibitor Nx-Nitro-L-arginine methyl ester hydrochloride (L-NAME, 100 μΜ) with or without nitrate for 24 h under the same conditions as described earlier.

### RNA and DNA extraction

Total RNA was extracted with RNAiso reagent (Takara, Dalian, China) according to the instruction of the manufacturer. The quantity and quality of the isolated total RNA was examined by spectrophotometry and gel electrophoresis, respectively.

Genomic DNA was isolated from the shoot of wild type (WT) and transgenic *Arabidopsis* plants according to the modified CTAB method^59^.

### Molecular cloning and sequence analysis of *SoHb*

According to the EST sequence of non-symbiotic hemoglobin of our previous SSH library, two different primers, Hb-R1 (5′-CAACGCGAATCTAGTCACCTC-3′) and Hb-R2 (5′-ACTTAACCACTAGAGCTTCCTGC-3′) were designed and the 5′ region was amplified according to the instruction manual (5′RACE System for Rapid Amplification of cDNA Ends, Version 2.0; Invitrogen, Carlsbad, CA).

According to the sequence of the internal conservative, and the 5′RACE product, two different primers, Hb-F1 (5′-AAAATGTCAACTGTTCTGCCTC-3′), Hb-F2 (5′-CATTTTGAGGTGACTAGATTCGC-3′) were designed. The 3′ region of the gene was amplified by a nested PCR. The first cycle was done with Hb-F1 and B26 (5′-GACTCGAGTCGACATCGATTTTTTTTTTTTTTTTTT-3′) under the following condition: template was firstly denatured at 94 °C for 5 min followed by 25 cycles of amplification (30 sec at 94 °C, 30 sec at 55 °C and 1 min at 72 °C) and by extension at 72 °C for 10 min. The amplified PCR product was diluted 10-fold and used as template in the second PCR using Hb-F2 and B26 under the following condition: 94 °C for 5 min, 30 cycles of 94 °C for 30 sec, 55 °C for 30 sec, 72 °C for 1 min and a final extension at 72 °C for 10 min. The PCR product was gel purified and cloned into the pMD18-T plasmid vector (TaKaRa, Dalian, China), and sequenced.

By comparing and aligning the EST sequence, the 5′ RACE and the 3′ RACE product, the full-length cDNA sequence was obtained. The open reading frame (ORF) sequence was amplified via PCR using a pair of primers HB-FB (5′-ggatcc ATGAGTCTCGAAAATGTCAAC-3′, *BamH* I site underlined) and HB-RX (5′-ctcgagATGAACTTCTAAAATTGTC-3′, *Xhol* I site underlined) following the condition: 94 °C for 5 min, 30 cycles of 94 °C for 30 sec, 55 °C for 30 sec, 72 °C for 1 min and a final extension at 72 °C for 10 min. The PCR product was gel purified and cloned into the pMD18-T plasmid vector (Takara, Dalian, China), and sequenced.

DNA sequence data was analyzed using the National Center of Biotechnology (NCBI) web site (http://www.ncbi.nlm.nih.gov). The BLAST program was used to search for sequence homology. The protein theoretical molecular weight and isoelectric point prediction were performed using DNAStar software. The alignment of the deduced protein sequences was computed using the DNAMan software employing standard parameters. The phylogenetic tree was computed using the NJ method in the MEGA 6.

### Expression analysis by qRT-PCR and semi-quantitative RT-PCR

For qRT-PCR, reverse transcription of RNA was carried out according to the instruction of the SYBR^®^ PrimeScript™ RT-PCR Kit II (Takara, Dalian, China). qRT-PCR was performed using the iCycler iQ Real-time PCR detection system (Bio-Rad, Hercules, CA). A dissociation curve was generated at the end of each PCR cycle to verify that a single product was amplified using software provided with the iCycler iQ real-time PCR detection system. To minimize sample variations, mRNA expression of the target gene was normalized relative to the expression of the house-keeping gene of spinach *18SrRNA* or *Arabidopsis Ubq1*. Three replicates were run for each sample. Primers used in qRT-PCR analyses were listed in Table S1.

Semi-quantitative RT-PCR was employed to analyze the transcript levels of *SoHb* in transgenic *Arabidopsis* plants and the expression of flower related genes. The reaction solutions and programs of RT-PCR were the same as those for *SoHb* isolation and thermal cycles (30). In addition, the cDNAs were amplified by *ACTIN* with the same procedure so as to confirm the loading of equal amounts of total RNA. RT-PCR experiments were repeated three times and the PCR products were detected by 1% agarose gel. Primers used in semi-quantitative RT-PCR analyses were listed in Table S2.

### SDS-PAGE and immunological analysis

Soluble proteins of plants were extracted from frozen material in a 25 mM Tris-HCl buffer (pH 7.6) with 1 mM MgCl_2_, 1 mM EDTA and a cocktail of protease inhibitors (aprotinin 5 mg mL^−1^, leupeptin 2 mg mL^−1^, pepstatin 0.1 mg mL^−1^, PMSF 1 M, Na_3_VO_4_ 1 mM, NaF 5 mM). After denaturisation, equal amounts of protein (30 mg) were separated on an SDS-polyacrylamide gel (12% (v/w) polyacrylamide). Proteins were then transferred onto a PVDF membrane (Bio-Rad, Hercules, CA, USA).

After incubation with a rabbit polyclonal anti-SoHb antibody (1:10,000), proteins were detected using a goat peroxidase-conjugated anti-rabbit antibody (1:4,000; Sigma) and visualized using ECL chemiluminescence (Bio-Rad, Hercules, CA). The secondary antibody was peroxidase-conjugated goat anti-rabbit IgG (1:5,000) (Santa Cruz Biotechnology, Inc.).

### Measuring of endogenous NO

Endogenous NO in spinach and *Arabidopsis* root after different treatment was visualised using the fluorescent probe, 4-amino, 5-aminomethyl-2’,7’-difluorescein diacetate (DAF-FM DA, Sigma-Aldrich, St. Louis, Mo, USA)^60^. Root tips of 4-week-old spinach or 2-week-old *Arabidopsis* were loaded with 10 μM DAF-FM DA in 20 mM HEPES buffer, pH 7.4, for 30 min, washed three-times in fresh buffer for 15 min. The root tips were then examined with a confocal lasers scanning microscope system (Nikon), using standard filters and collection modalities for DAF-FM DA green fluorescence (excitation 485 nm; emission 515 nm). Treatments were repeated 5 times. Signal intensities of green fluorescence in the images were quantified using Image J software (http://rsb.info.nih.gov/ij/).

### Binary expression vector construction and *Arabidopsis* transformation

The coding sequence of *SoHb* was amplified with the primer pair Hb-FB and Hb-RX, and subcloned into gateway entry vector pENTR-2B (VIB, Gent, Belgium). The destination vector was pK7m34GW2–8m21GW3 (VIB, Gent, Belgium). The Gateway LR Clonase plus Enzyme Mix (Invitrogen, Carlsbad, CA) was used to perform the LR reactions according to the manufacturer’s instructions to obtain the pK2GW7-*SoHb*. The reaction mixture was incubated overnight at 25 °C and transformed into *E. coli* DH5*a* competent cells. The recombinant clones were selected on LB-medium plates with 50 μg mL^−1^ spectinomycin. The constructs were introduced into *Agrobacterium tumefaciens* strain EHA105 by electroporation and transformed into WT *Arabidopsis* (Columbia ecotype) by the floral-dip method^61^.

The T1 seeds were germinated on half-strength Murashige and Skoog (MS) agar plates containing kanamycin (30 mg L^−1^). The T2 seeds were germinated on half-strength MS agar plates containing kanamycin (50 mg L^−1^), and the resistant plants were transferred to nursery soil to obtain homozygous T3 seeds. Then the genomic DNA PCR, RT-PCR and western blot confirmed the presence and expression of SoHb in transgenic *Arabidopsis*. The T3 homozygous lines were used for further physiological analyses.

### Stress tolerance assays for *Arabidopsis*

The WT and T3 progeny resulting from self-fertilization of *Arabidopsis* transgenic plants seeds were surface-sterilized and germinated in sterile plates containing MS salt, 3% (w/v) sucrose, and 0.7% (w/v) agar, pH 5.8. To determine the effect of nitrate stress, germinated seeds were moved to MS medium supplemented with 100 and 200 mM nitrate (normal 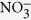 concentration in the MS medium of 40 mM was used as a control). To determine the effect of NO tolerance of transgenic plants, 0, 30, 50, and 150 μM SNP or GSNO was added into the MS medium. To investigate if exogenous SNP could improve the nitrate stress tolerance of transgenic plants, 5μM SNP was added into the MS medium supplemented with 100 mM nitrate. The seeds were geminated in controlled environment chambers at an irradiance of 140 μmol photons m^−2^ s^−1^, 22 °C and 60% relative humidity in a photoperiod of 8 h/16 h light/dark regime. Plates were placed vertically on shelves to facilitate comparison of root growth.

For seedling treatment, 12 seedlings each of 2-week-old WT and two transgenic *Arabidopsis* lines were grown in the same pot (3 pots per experiments; 3 repeats) with regular watering every 2 days, followed by watering every 2 days with a 100 mM nitrate solution for 15 days. At the end of the treatment, photographs were taken and physiological parameters were analyzed.

### Lipid peroxidation assays

Lipid peroxidation of WT and transgenic *Arabidopsis* seedling shoots was determined after 100 mM nitrate treatment for 15 d by estimating the formation of malondialdehyde (MDA), a product of lipid peroxidation, using the thiobarbituric acid reaction method^62^.

### Antioxidant enzymes activities assays

The transgenic seedlings after 100 mM nitrate treatment for 15 d were used for the enzyme analysis. 0.2 g of shoot tissues was homogenized in 2 mL of 0.05 M sodium phosphate buffer (pH 7.8, containing 1.0 mM EDTA, and 2% (w/v) PVP). The homogenate was centrifuged at 10,000 × *g* for 20 min at 4 °C, and the supernatant was used for all enzyme activity assays. All steps in the preparation of each enzyme extract were carried out at 4 °C. All spectrophotometric analyses were conducted using UV-2450PC spectrophotometer (Shimadzu, Tokyo, Japan).

SOD activity was assayed by measuring its ability to inhibit the photochemical reduction of nitroblue tetrazolium (NBT) spectrophotometrically at 560 nm^62^. The reaction mixture consisted of 0.3 mL each of 0.75 mM NBT, 130 mM methionine, 0.1 mM EDTA-Na_2_, 0.02 mM riboflavin, sterilized water, and 1 mL of 50 mM sodium phosphate buffer (pH 7.8). The reaction was started by adding 0.5 mL of enzyme extract and carried out for 20 min at 25 °C under a light intensity of 300 μmol m^−2^ s^−1^. One unit of enzyme activity was defined as the quantity of SOD required to produce 50% inhibition of reduction of NBT.

CAT activity was measured as the decline in absorbance at 240 nm due to the decline of extinction of H_2_O_2_. The reaction mixture containing 25 mM sodium phosphate buffer (pH 7.0), 10 mM H_2_O_2_ and 0.1 mL enzyme extract. The reaction was started by adding H_2_O_2_^63^.

### Chlorophyll fluorescence assays

Chlorophyll fluorescence was analyzed with PAM (Pulse-Amplitude-Modulation) Chlorophyll Fluorometer (Heinz-Walz-GmbH, Effeltrich, Germany). The measurements of the maximal quantum yield of PSII (Fv/Fm) were done according to Bai *et al.*^64^.

### Analysis of flowering time

WT and transgenic *SoHb* lines were grown in soil under 16 h light/8 h dark cycles and photographed after 28 and 33 d of growth. Flowering time was scored as the number of rosette leaves and days to flowering at the stage when the first flower appeared in wild-type and transgenic *SoHb* lines.

### Statistical analysis

Values presented were means ± one standard deviation (SD) of three replicates. Statistical analysis was performed by analysis of variance (ANOVA) using DPS software. Difference between treatments was separated by the least significant difference (LSD) test at a 0.05 probability level. For statistical tests, each transgenic line was only compared to WT.

## Additional Information

**How to cite this article**: Bai, X. *et al.* Overexpression of spinach non-symbiotic hemoglobin in *Arabidopsis* resulted in decreased NO content and lowered nitrate and other abiotic stresses tolerance. *Sci. Rep.* **6**, 26400; doi: 10.1038/srep26400 (2016).

## Supplementary Material

Supplementary Information
