# Supplementary material for: Overexpression of spinach non-symbiotic hemoglobin in Arabidopsis resulted in decreased NO content and lowered nitrate and other abiotic stresses tolerance
Source: Sci Rep. 2016 May 23;6:26400. doi: 10.1038/srep26400 (PMC4876387; doi:10.1038/srep26400)

Title page

Overexpression of spinach non-symbiotic hemoglobin in *Arabidopsis* resulted in decreased NO content and lowered nitrate and other abiotic stresses tolerance

Xuegui Bai[+], Juan Long[+], Xiaozhao He, Jinping Yan, Xuanqin Chen, Yong Tan, Kunzhi Li, Limei Chen & Huini Xu*

Faculty of Life Science and Technology, Kunming University of Science and Technology, Jingming South Street, Kunming, Yunnan 650224, P.R. China

[+]These authors contributed equally to this work.

Xuegui Bai e-mail: baixuegui@126.com
Juan Long e-mail: longjuan63@126.com
Xiaozhao He e-mail:he.xiaozhao@163.com
Jinping Yan e-mail:jpyan2015@163.com
Xuanqin Chen e-mail: chenxuanqin12@aliyun.com
Yong Tan e-mail: 57221031@qq.com
Kunzhi Li e-mail:likunzhi63@126.com
Limei Chen e-mail:chenlimeikm@126.com
*Huini Xu (Correspondence author) e-mail:hnxusun@126.com

Supplementary information

Supplementary Table S1 Primers for the qRT-PCR

primer	forward	reverse
Ubq1	AACTTTGGTGGTTTGTGTTTTGG	TCGACTTGTCATTAGAAAGAAAGAGATAA
SoHb	AGCCCATGCCATGTCTGTTT	GGCCTCCTTTATCGTTTCCA
MnSOD	CAGAAGCATCACCAGGCTTA	CGAATGGTTGACATGACCTC
CAT1	GTCGGATGAGGAAGCTATCAG	TCCATCACCTGAACGAAGAG
RD22	CATGAGTCTCCGGGAGGAAGTG	CG GCTGGGGTAAAGA AGTTGTC
RD29A	GATGGAAGATTCTGTCTCAACGAT	GTTTCTCCTTCACTATCTCCTCCG
DREB2A	GTGGAGTGGAGCCGATGTATTG	GAATCCTGCTGTTGTTGCTGAC
P5CS1	AAGGCTTGTGATACGGATATGG	ATGCACAAGAAGGGTTTCCA
18SrRNA	CCATAAACGATGCCGACCAG	AGCCTTGCGACCATACTCCC

Supplementary Table S2 Primers for the semi-quantitative RT-PCR

primer	forward	reverse
CO	CACACCATCAAACTTACTACATCTG	CTGAAAATTCTGTTGGTTATGGCAC
SOC1	ATGGTGAGGGGCAAAACTCAGATGAA	TTCATGAGATCCCCACTTTTCAGAGA
FLC	ATGGGAAGAAAAAAACTAGAAATCAA	CTAATTAAGTAGTGGGAGAGTCAC
GI	CTTGTTGATGGAGAAGCTTGTACAT	GCTTATTGGGACAAGGATATAGTAC
ACTIN	CTTCTTCCGCTCTTTCTTTCCAAG	GAGCTTCTCCTTGATGTCTCTTAC
SoHb	AAAATGTCAACTGTTCTGCCTC	CAACGCGAATCTAGTCACCTC

Supplementary Figure S1 Nucleotide and deduced amino acid sequences of the *SoHb* cDNA. The cDNA sequence included the putative coding region and the 5′ and 3′ non-coding regions. The amino acid of the putative coding region was shown beneath the DNA sequence. Stop codon (TGA) was marked with an asterisk.

```
1     ATATTACCTTTTATCTTAGCAAAGTGATTCAGATTTTTGCATAAATCAACCATGAGTCTC
1                                                        M  S  L
61    GAAAATGTCAACTGTTCTGCCTCAAACGTGTTTACAGAAGAGCAGGAAGCTCTAGTGGTT
4      E  N  V  N  C  S  A  S  N  V  F  T  E  E  Q  E  A  L  V  V
121   AAGTCATGGAATGTCATGAAAAAGAACTCTGCTGAATTGGGTCTCAAGTTGTTCTTGAGG
24     K  S  W  N  V  M  K  K  N  S  A  E  L  G  L  K  L  F  L  R
181   ATCTTTGAGATTGCACCAACGGCTAAGAAAATGTTCTCTTTTCTAAGGGACTCAAACGTT
44     I  F  E  I  A  P  T  A  K  K  M  F  S  F  L  R  D  S  N  V
241   CCCTTGGAGCAGAACCCTAAGCTCAAAGCCCATGCCATGTCTGTTTTCGTCATGACTTGT
64     P  L  E  Q  N  P  K  L  K  A  H  A  M  S  V  F  V  M  T  C
301   AAATCAGCTGTTCAGCTTAGAAAATCCGGCAAGATCACGGTTGGAGAGTCTAGCTTAAAG
84     K  S  A  V  Q  L  R  K  S  G  K  I  T  V  G  E  S  S  L  K
361   CATATGGGGTCTGTCCACGTGAAATATGGAGTTGTTGATGAACATTTTGAGGTGACTAGA
104    H  M  G  S  V  H  V  K  Y  G  V  V  D  E  H  F  E  V  T  R
421   TTCGCGTTGTTGGAAACGATAAAGGAGGCCGTACCAGAGATGTGGTCGCCGGAGATGAAG
124    F  A  L  L  E  T  I  K  E  A  V  P  E  M  W  S  P  E  M  K
481   AATGCTTGGGCTGAAGCTTTTAATCAGTTGGTTGCTGCTATTAAGGCTGAAATGAAGCCT
144    N  A  W  A  E  A  F  N  Q  L  V  A  A  I  K  A  E  M  K  P
541   AAATCTCAAGCTTAAGTTCACTTGTAAAAAATATTGGTTTTGACAATTTTAGAAGTTCAT
164    K  S  Q  A  *
601   TTAAGAATAAATAAATACAAGTACTATCAATGTATACATACGTCACACCAAAAAAAAAAA
661   AAAAAAA
```

Supplementary Figure S2 The NO fluorescence analysis of spinach root under nitrate stress supplemented with NO donor, NO scavenger or inhibitor by confocal laser microscope. (a) NO content in spinach root without treatment. (b) NO content in spinach root with SNP treatment. (c) NO content in spinach root with nitrate treatment. (d) NO content in spinach root with SNP and nitrate treatment. (e) NO content in spinach root with tungstate and nitrate treatment. (f) NO content in spinach root with L-NAME and nitrate treatment. (g) NO content in spinach root with cPTIO and nitrate treatment. (h) NO content in spinach root with SNP, cPTIO and nitrate treatment. Bars=100 μM.

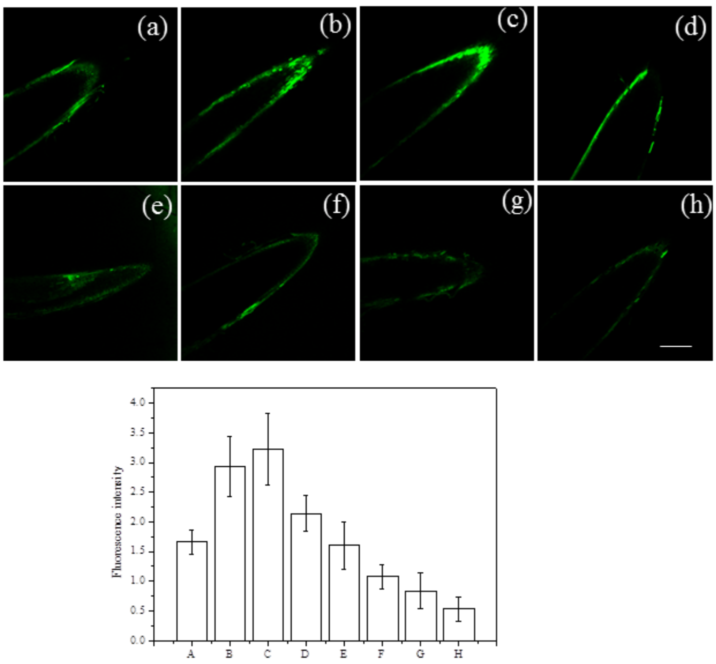

Supplementary Figure S3 Characterization of *Arabidopsis* transgenic lines overexpression *SoHb*. (a) Schematic diagram of the gene cassettes used for *Arabidopsis* transformation. (b), (c), (d) were analysis of transgenic and WT plants by PCR, RT-PCR and western blot, respectively. M: Marker. P: Plasmid of pK2GW7-*SoHb*. Hb-1, 2, 3, 5, 6, 7, 9 are different transgenic lines. The *Arabidopsis* *ACTIN* was used as an endogenous control.

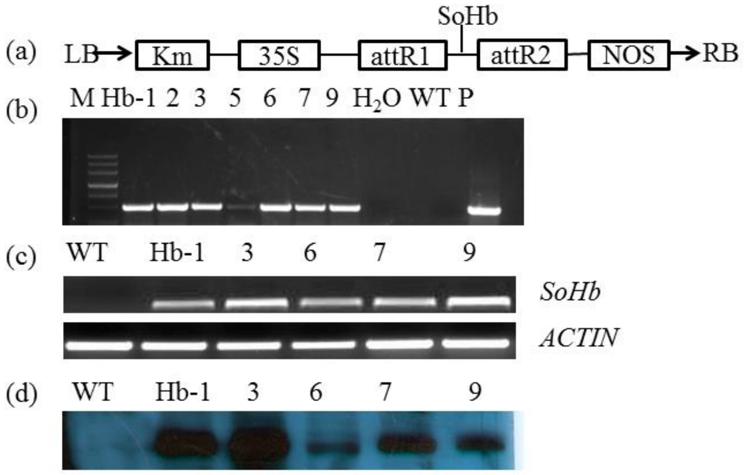

Supplementary Figure S4 Effect of excess GSNO on the growth of *SoHb* transgenic *Arabidopsis* for 7 days. Seedling morphology (a), root length (b), fresh weight (c) of the WT and transgenic lines grown on MS supplemented with 0, 30, 50, and 150 μM GSNO. Bars=1cm.

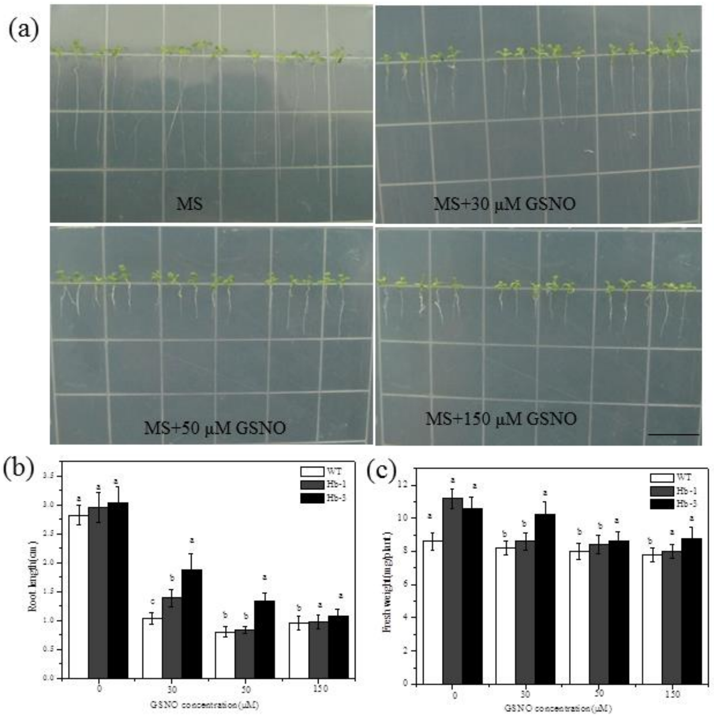

Supplementary Figure S5 Effect of exogenous SNP on the growth of nitrate-stressed *SoHb* transgenic *Arabidopsis* for 12 days. Seedling morphology of plant grown on MS medium(a), MS medium added 100 mM nitrate (b), MS medium added 5 μM SNP(c) and MS medium added 5 μM SNP and 100 mM nitrate of the WT and transgenic lines plants. Bars=1cm.

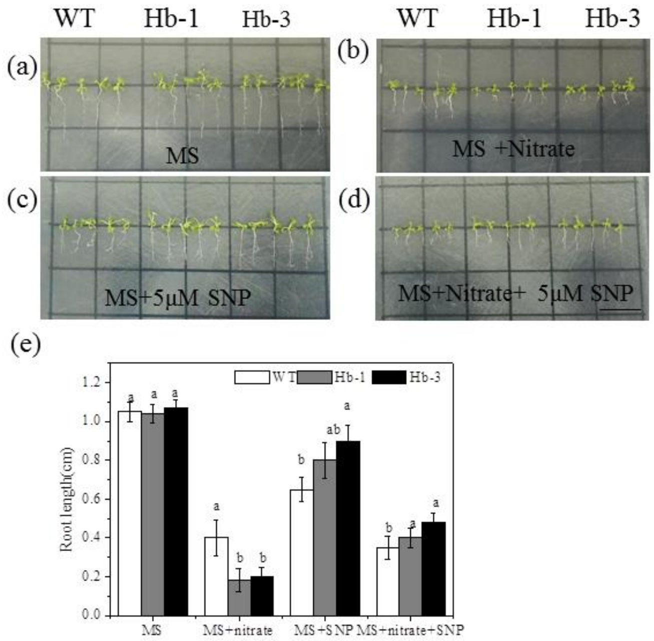

Supplementary Figure S6 Comparison of root development in *SoHb* overexpression transgenic and WT plants. (a)The growth of WT, Hb-1, Hb-3 on the MS medium for 2 weeks. (b) The lateral root number of WT, Hb-1, Hb-3.

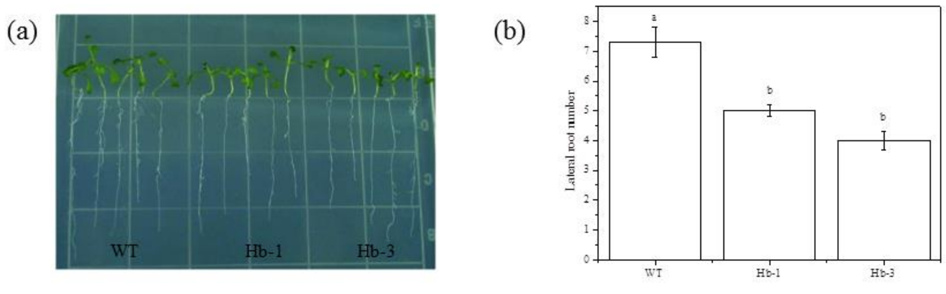

Supplement: Supplementary Information [file srep26400-s1.pdf]
